# The global impact of the COVID-19 pandemic on the prevention, diagnosis and treatment of hepatitis B virus (HBV) infection

**DOI:** 10.1136/bmjgh-2020-004275

**Published:** 2021-01-05

**Authors:** Caitlin M Pley, Anna L McNaughton, Philippa C Matthews, José Lourenço

**Affiliations:** 1 School of Clinical Medicine, University of Cambridge School of Clinical Medicine, Cambridge, UK; 2 Nuffield Department of Medicine, Medawar Building for Pathogen Research, University of Oxford, Oxford, UK; 3 Department of Microbiology and Infectious Diseases, Oxford University Hospitals NHS Foundation Trust, Oxford, UK; 4 Department of Zoology, University of Oxford, Oxford, UK

**Keywords:** viral hepatitis, SARS, public health, prevention strategies

## Abstract

The COVID-19 pandemic caused by the SARS-CoV-2 virus has resulted in a myriad of interventions with the urgent aim of reducing the public health impact of this virus. However, a wealth of evidence both from high-income and low-income countries is accruing on the broader consequences of such interventions on economic and public health inequalities, as well as on pre-existing programmes targeting endemic pathogens. We provide an overview of the impact of the ongoing COVID-19 pandemic on hepatitis B virus (HBV) programmes globally, focusing on the possible consequences for prevention, diagnosis and treatment. Ongoing disruptions to infrastructure, supply chains, services and interventions for HBV are likely to contribute disproportionately to the short-term incidence of chronic hepatitis B, providing a long-term source of onward transmission to future generations that threatens progress towards the 2030 elimination goals.

**Summary box**

- The maintenance of programmes to tackle hepatitis B virus (HBV) is fragile, particularly in low-income and middle-income countries, as funding is frequently not embedded in domestic budgets and programmes are often not supported by national strategic plans.
- The most vulnerable members of society are more likely to be affected by HBV, and the COVID-19 pandemic is amplifying pre-existing economic and public health inequalities.
- Historical data clearly illustrate the disruptive legacy of political and economic crises on HBV vaccination programmes. Evidence is slowly emerging on the greater health impacts of COVID-19 public health interventions on current HBV initiatives, both in high-income and low-income countries, with disruptions to infrastructure, services and supply chains for diagnosis, vaccination and treatment recorded.
- Ongoing short-term disruptions to HBV programmes can have strong repercussions on early childhood incidence, fuelling an increase in the global burden of chronic infection in the long term and providing a source of onward transmission to future generations that threatens progress towards the 2030 elimination goals.

## Background

The COVID-19 pandemic, which by October 2020 had caused more than a million deaths worldwide,^1^ has exposed fault-lines and vulnerabilities in global health systems. The human and economic costs of the pandemic extend far beyond the direct impact of SARS-CoV-2 infection, including death and illness due to health system disruptions, as well as unemployment and poverty at a massive scale. We here consider the impact of the COVID-19 pandemic on hepatitis B virus (HBV), a blood-borne virus that accounts for a large global burden of chronic liver disease, resulting in over one million deaths every year from liver cancer and liver cirrhosis,^2 3^ despite the availability of an effective vaccine and suppressive antiviral treatment.

HBV is highly endemic in some of the world’s poorest populations with limited healthcare infrastructure, particularly in sub-Saharan Africa and Asia Pacific.^4^ Just 20 countries account for over 75% of the global burden of HBV.^3^ It is estimated that between 1990 and 2020, childhood vaccination campaigns have prevented 310 million new HBV infections,^3^ but other high risk groups remain vulnerable to infection, including people who inject drugs and men who have sex with men.^3^ The Sustainable Development Goals set ambitious targets, aiming to eliminate HBV infection as a public health problem by 2030. A multi-pronged approach will be needed to deliver these aims,^2^ including the prevention of vertical transmission and universal access to testing and treatment services. Evidence of the effect of the COVID-19 pandemic on the estimated 290 million people living with chronic HBV (CHB) worldwide has progressively accrued from primary research studies using physician surveys and routine health system data, with reports that approximately 90% of viral hepatitis services have been disrupted during the COVID-19 pandemic.^5 6^ Major global public health interventions will remain essential to curtailing the pandemic, but awareness of broader consequences is crucial. This article provides an evidence synthesis of the pandemic’s global impact on HBV prevention, diagnosis and treatment, and suggestions for domains where action is needed to address specific challenges.

## Disrupted vaccination campaigns

**Figure 1:**
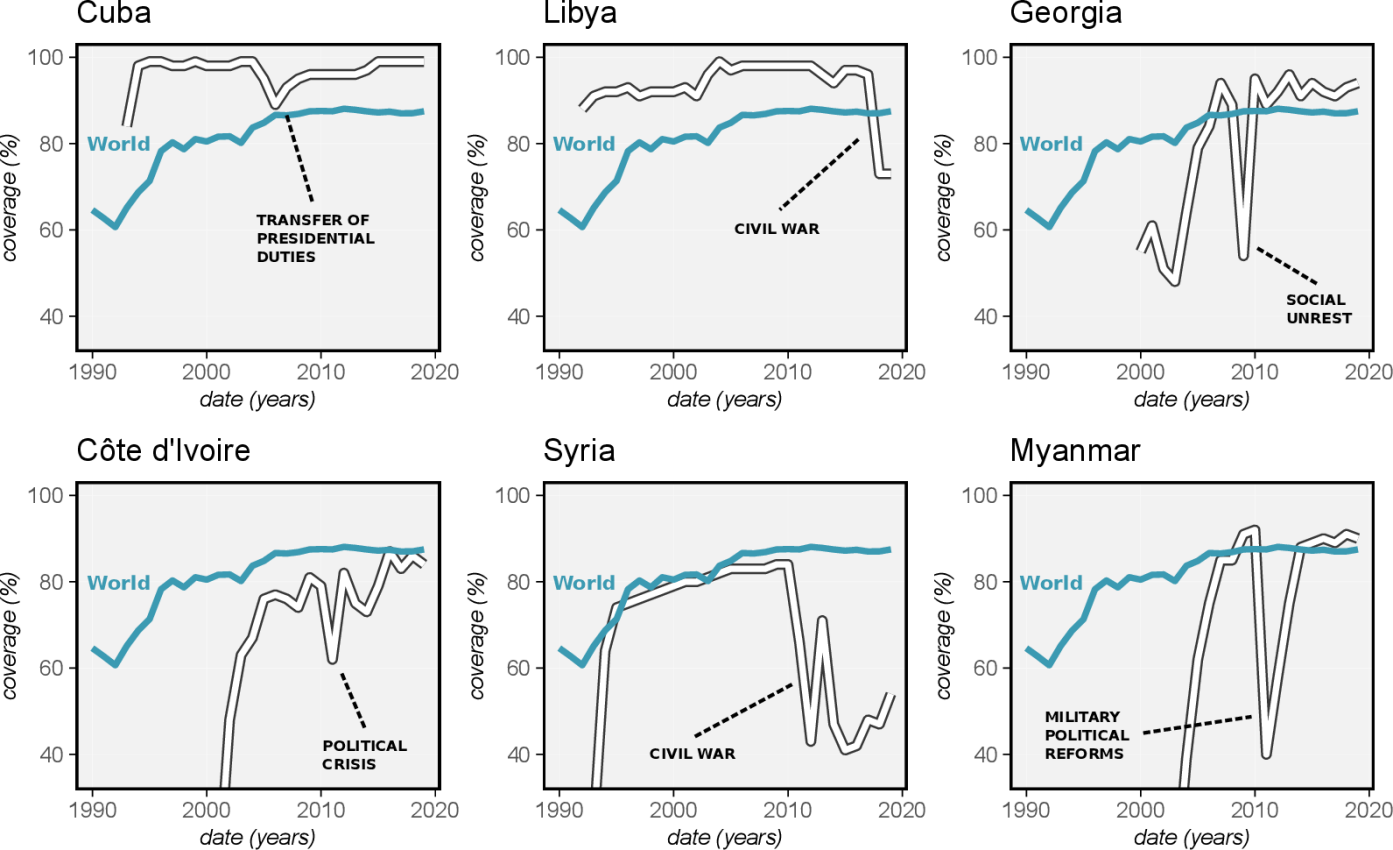
Hepatitis B vaccination coverage annotated to show correlation with societal disruption. Panels show the temporal correlation of drops in vaccination coverage with national crises (white line), such as the transfer of presidential duties in Cuba (2006–2008), the civil war in Libya (2014–present), a period of social unrest in Georgia (2009), a recent Ivorian political crisis (2010–2011), the civil war in Syria (2011–present) and the period of military-enforced political reforms in Myanmar (2011–2015). Data source: WHO/UNICEF (apps.who.int/immunization_monitoring/globalsummary).

There is previous evidence to show that routine immunisation programmes are highly vulnerable to disruption resulting from epidemics, political upheaval or economic crises. When vaccination coverage rates sharply dipped in West Africa during the 2013–2016 Ebola outbreak, the incidence of measles rapidly rebounded.^7^ Although HBV global vaccination coverage has steadily increased since the 1990s, previous experience shows correlation of declines in vaccination coverage with political and economic unrest that disrupt infrastructure (figure 1). Since the progression to overt liver disease occurs slowly, the impact of a drop in HBV vaccination coverage may go unnoticed for decades in settings without adequate diagnostic infrastructure.

Preliminary data from the Institute for Health Metrics and Evaluation indicate that overall global vaccination coverage levels in 2020 have dropped to levels last seen in the 1990s, threatening 25 years of progress in just 6 months.^8^ The USA’s federally financed ‘Vaccines for Children’ Programme has documented notable declines in vaccine ordering and administration after declaration of the national emergency on 13 March 2020, although more markedly in children older than 24 months than younger children, reflecting some success in maintaining routine vaccination of infants.^9^ In England, electronic health records have shown that coverage of the measles, mumps, rubella vaccination dropped by 19.8% when physical distancing measures were implemented between February and April 2020, compared with the same period in 2019.^10^

Reduced vaccination coverage may have particularly strong repercussions on HBV incidence in infancy and early childhood, contributing to an increase in the global burden of chronic infection and providing a long-term source of onward transmission that threatens progress towards the 2030 elimination goals. The repercussions of the COVID-19 pandemic on HBV vaccination and control may even outweigh the number of direct COVID-19 deaths in the long term. A recent model has projected that for one excess COVID-19 death attributable to visiting a vaccination delivery point, mostly in the older household contacts of children, the deaths of 84 children under 5 years could be prevented if routine childhood immunisation programmes were sustained in sub-Saharan Africa.^11^

Sustaining HBV vaccination is further complicated by pandemic-driven bottlenecks in the supply chain of vaccines, increases in home births hindering administration of birth dose vaccine, changes in healthcare seeking behaviour and potential effects on vaccine hesitancy.^12^ Vaccination acceptance is closely linked to fear of the related disease and trust in government agencies.^13^ Both of these factors are labile due to COVID-19. While risk perception of the danger of infectious diseases may have increased, the politicisation of the pandemic has also caused a surge in anti-science sentiment and government distrust across the globe.

## Altered transmission dynamics

Evidence on the effect of the pandemic on the transmission of HBV is limited. While transmission may have decreased due to physical contact and movement restrictions imposed in many countries, these exceptional circumstances may have also led to an increase in risk behaviours for HBV transmission, including alcohol and drug use, unprotected sex, reduced anti-viral treatment availability and increased home births.^12^ Disruption of harm reduction services, such as needle exchange and opioid substitution therapy, which are already scarce in many low-income and middle-income countries (LMICs), not only jeopardises the health of people who inject drugs and increases the risk of overdoses, but also increases the risk of transmission of blood-borne viral infections. COVID-19 restrictions leading to temporary closures of health centres offering harm reduction services have been reported from South Africa,^14^ and although data in other LMICs are lacking, similar events are likely to be widespread.

Vertical transmission may also increase as antenatal care services are disrupted and more women give birth at home, risking an increased burden of CHB among infants born during the pandemic. Reduced access to interventions for prevention of mother to child transmission (PMTCT) (including antenatal diagnostics, antiviral therapy, hepatitis B immunoglobulin and vaccine birth doses) endangers a generation of individuals, who are more likely to develop chronic infection following exposure early in life. In the long term, such temporary increases in HBV chronic prevalence in the young can contribute to a positive feedback loop of transmission to subsequent generations.

## Decreased diagnostic capacity

Even before COVID-19, only approximately 1% of viral hepatitis cases were diagnosed in sub-Saharan Africa.^12^ Therefore, although the pandemic may further exacerbate difficulties in identifying infections, in some parts of the world, the impact of disrupted vaccination campaigns, PMTCT programmes and antiviral treatment on the HBV epidemic will likely be greater than reduced diagnostic capacity. Nonetheless, missed diagnoses prevent entry into care and can have a long-term impact on transmission dynamics. Access to healthcare, including testing and screening programmes, has been affected by the diversion of funding, human resources and infrastructure to the COVID-19 response, as well as reduced demand as a result of movement restrictions and fear of contracting COVID-19 in healthcare settings.^1^ In sub-Saharan Africa, a decrease in new HBV diagnoses was observed even before the first local COVID-19 cases were reported, suggesting an early influence of outbreaks in other countries.^4^ In April, health centres in Burkina Faso, the Gambia and Tanzania recorded significant declines in new patients reviewed in outpatient clinics compared with the start of the year, falling by 71%, 83% and 95% respectively, with the primary reason cited as patients’ fear of entering health services.^4^ Disruptions in global supply chains, particularly early on in the pandemic, affected the availability of diagnostic platforms and reagents, and shortages of nucleic acid tests for HBV DNA testing were reported from sub-Saharan Africa.^4^

## Reduced access to treatment

Clinical guidelines recommend that HBV therapy should be initiated and continued regardless of COVID-19 status.^15^ However, access to healthcare has been constrained for HBV-positive individuals as a result of a myriad of interdependent factors, including redirected finances, redeployment of health workers, closure of facilities, supply chain disruptions, movement restrictions, fear of entering health facilities and the impracticality of telemedicine in many settings.^12^ At the height of Italy’s first wave, one in four hepatology wards had been converted into COVID-19 wards and the services of one in four outpatient hepatology clinics had been completely interrupted.^16^ The degree of disruption in Italy was negatively correlated with the severity of HBV-associated disease, but nevertheless only 18% of patients with hepatocellular carcinoma and 32% of patients with decompensated cirrhosis experienced continuity of service delivery.^16^ The initiation of drug treatment for HBV was also postponed in 23% of centres in Italy.^16^ Reduced demand for healthcare has been reported from LMICs as well as high-income countries (HICs).^4^ In Japan, Singapore and the USA, consultations for CHB decreased significantly compared with 2019, with the largest drop in adults older than 65 years.^17^

In a survey conducted by the World Hepatitis Alliance, 52% of frontline health workers in LMICs reported that patients on treatment for viral hepatitis were unable to access medication.^6^ The primary reasons cited by the respondents were patient anxiety surrounding health facilities, national stay-at-home orders and insufficient financial resources to purchase medicines out-of-pocket as pandemic job losses impact household incomes.^6^ Barriers to accessing HBV treatment were further compounded by supply chain disruptions of antiviral drugs in both the public and private sectors in sub-Saharan Africa.^12^ Interrupted, prematurely terminated or substandard treatments increase the risk of disease flare with public health consequences, including increased transmission risk and the threat of drug resistance emergence.

While emerging evidence suggests a link between cirrhosis and end-stage liver disease and the risk of severe COVID-19,^18 19^ the control of HBV is further complicated by the small risk of reactivation of dormant infection following treatment with tocilizumab and corticosteroids for COVID-19.^15 20^ This risk may be lowered with short courses of antiviral prophylaxis alongside COVID-19 medications in patients with comorbid CHB, however, this approach would require the necessary healthcare infrastructure to maintain diagnostic and treatment services.^15 20^

## Health inequalities

The COVID-19 pandemic is amplifying existing economic and public health inequalities. The pandemic is likely to cause a worldwide recession and the contraction of national economies, pushing millions more below the poverty line, especially in countries without universally accessible health systems and already high levels of out-of-pocket spending on health.^21^ Furthermore, the most vulnerable members of society are not only more likely to be affected by HBV, but they are also more likely to have comorbid non-communicable diseases that raise the risk of a severe COVID-19 disease course. Rural and indigenous communities in LMICs, as reported in India and Nigeria, are most severely affected by movement restrictions, as they impede access to health centres and the ability to earn a living wage.^6^ Individuals working in unstable employment arrangements risk losing their health insurance, and those working in the informal sector frequently already pay for healthcare expenditures out-of-pocket, with no access to fiscal stimulus packages and other social safety nets if they lose their employment. Such issues extend to HICs, such as the USA, where a large number of job losses have led to 6.2 million people losing their health insurance since the onset of the COVID-19 pandemic.^22^ Drops in healthcare coverage will likely lead to worsening of chronic conditions, including CHB, and may reduce the incentive to seek testing services when treatment is unaffordable.

## Conclusion

**Figure 2:**
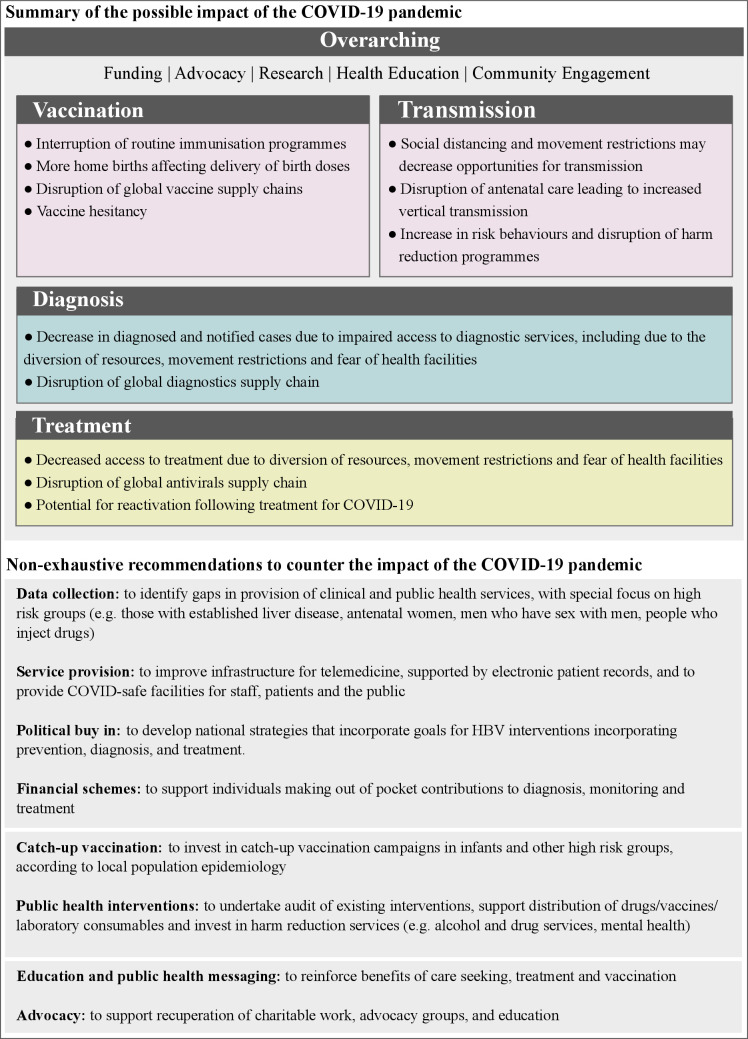
Top panel: summary of the possible impact of the COVID-19 pandemic on HBV vaccination, transmission, diagnosis and treatment. Overarching factors, including the availability of funding and the ability to conduct advocacy, research, health education and community engagement, affect all levels of HBV control. Bottom panel: table of recommendations for policymakers and for further research to better understand and mitigate the impact of COVID-19 on HBV prevention, diagnosis and treatment. HBV, hepatitis B virus.

Beyond prevention, diagnosis and treatment (figure 2), the impact of COVID-19 on the global health architecture and development assistance programmes may also have far-reaching effects on global efforts to control HBV. The maintenance of viral hepatitis programmes is fragile, as funding is frequently not embedded in domestic budgets and programmes are often not supported by national strategic plans. The diversion of funding is also impeding the ability of civil society to continue their advocacy activities and engage in health education activities in communities affected by hepatitis. Disruption of HBV prevention, diagnosis and treatment services threatens to derail progress made so far on reducing the global burden of HBV, and pushes the 2030 goal of elimination further out of reach. Building on the findings of this study and the WHO pulse survey on continuity of essential health services during the COVID-19 pandemic,^23^ we provide a non-exhaustive suite of recommendations (figure 2) for policymakers and researchers to better understand and mitigate the impact of the COVID-19 pandemic on HBV prevention, diagnosis and treatment.

## Data Availability

There are no data in this work.
